# Improvement of Non-motor Symptoms and Quality of Life After Deep Brain Stimulation for Refractory Dystonia: A 1-Year Follow-Up

**DOI:** 10.3389/fneur.2021.717239

**Published:** 2021-10-04

**Authors:** Clarice Listik, Rubens Gisbert Cury, Sara Carvalho Barbosa Casagrande, Eduardo Listik, Debora Arnaut, Natally Santiago, Valquiria Aparecida Da Silva, Ricardo Galhardoni, Júlia de Lima Arantes Machado, Jessica Campelo de Almeida, Egberto Reis Barbosa, Manoel Jacobsen Teixeira, Daniel Ciampi De Andrade

**Affiliations:** ^1^Department of Neurology, School of Medicine, University of São Paulo, São Paulo, Brazil; ^2^Department of Pathology, University of Alabama at Birmingham, Birmingham, AL, United States

**Keywords:** dystonia, deep brain stimulation, non-motor symptoms, pain, quality of life

## Abstract

**Introduction:** Deep brain stimulation (DBS) is a treatment option for refractory dystonia's motor symptoms, while its non-motor symptoms (NMS) have been less systematically assessed. We aimed to describe the effects of DBS on NMS in refractory generalized inherited/idiopathic dystonia prospectively.

**Methods:** We evaluated patients before and 1 year after DBS surgery and applied the following scales: Burke–Fahn–Marsden Rating Scale (BFMRS), NMS Scale for Parkinson's Disease (NMSS-PD), Parkinson's Disease Questionnaire-8, short-form Brief Pain Inventory (BPI), Neuropathic Pain Symptom Inventory (NPSI), and short-form McGill Pain Questionnaire (MPQ).

**Results:** Eleven patients (38.35 ± 11.30 years) underwent surgery, all with generalized dystonia. Motor BFMRS subscore was 64.36 ± 22.94 at baseline and 33.55 ± 17.44 1 year after DBS surgery (47.9% improvement, *p* = 0.003). NMSS-PD had a significant change 12 months after DBS, from 70.91 ± 59.07 to 37.18 ± 55.05 (47.5% improvement, *p* = 0.013). NMS changes were mainly driven by changes in the gastrointestinal (*p* = 0.041) and miscellaneous domains (*p* = 0.012). Seven patients reported chronic pain before DBS and four after it. BPI's severity and interference scores were 4.61 ± 2.84 and 4.12 ± 2.67, respectively, before surgery, and 2.79 ± 2.31 (0.00–6.25) and 1.12 ± 1.32 (0.00–3.00) after, reflecting a significant improvement (*p* = 0.043 and *p* = 0.028, respectively). NPSI score was 15.29 ± 13.94 before, while it was reduced to 2.29 ± 2.98 afterward (*p* = 0.028). MPQ's total score was 9.00 ± 3.32 before DBS, achieving 2.71 ± 2.93 after (*p* = 0.028).

**Conclusions:** DBS improves NMS in generalized inherited/idiopathic dystonia, including chronic pain.

## Highlights

- DBS improves non-motor symptoms in generalized inherited/idiopathic dystonia.
- Chronic pain is improved after DBS in generalized inherited/idiopathic dystonia.
- Quality of life improvement was driven by the non-motor symptoms' improvement.

## Introduction

Dystonia is characterized by abnormal movements and postures (1). Regarding etiology, the dystonias may have acquired causes like infections, perinatal brain injury, neoplastic, and others; inherited through autosomal dominant, autosomal recessive, X-linked, and mitochondrial genes; and idiopathic causes (1). The monogenic forms have been designated, as mentioned before, for many years as DYT and a sequential number (e.g., DYT1, DYT6, and DYT16). Nowadays, a new classification system has been proposed and uses the gene implicated in the dystonia (e.g., DYT-*TOR1A*, DYT-*THAP1*, and DYT-*PRKRA*, respectively) (2). Generalized inherited/idiopathic dystonia is often refractory to pharmacological treatments. Deep brain stimulation (DBS) targeting the globus pallidus internus (GPi) or the subthalamic nucleus (STN) is already an established treatment, and beneficial motor outcomes have been extensively described (3–6). The acquired dystonias have scarcer literature (5). They usually do not respond as well to DBS when compared with the aforementioned group, except the tardive dystonias (5). Patients with generalized inherited/idiopathic dystonia usually have the best response to DBS treatment when compared with most other dystonia types, such as acquired forms of the disease (5). GPi-DBS and STN-DBS seem to have similar motor and quality of life outcomes in dystonia, with STN-DBS being more interesting due to better battery drainage (6, 7). However, dystonia non-motor symptoms (NMS), including pain and mood, have been less frequently investigated, and the detailed effects of DBS on NMS remain mainly unknown (8–10). Our main objective was to describe NMS outcomes after DBS surgery for refractory generalized inherited/idiopathic dystonia in a prospective exploratory study with a focus on chronic pain.

## Methods

This study was approved by our Institutional Ethics Review Board (protocol number #48607515.5.0000.0068), and all patients gave written informed consent before being included in the study. Patients were evaluated from December 2015 to July 2019.

### Patients

Patients had generalized dystonia of inherited/idiopathic etiology (1), and all underwent DBS surgery. Patients were excluded if they were younger than 18 years old, had other types of dystonia, or did not consent to participate (3, 4, 6).

### Study Design

This prospective observational study evaluated patients before and 1 year after DBS surgery, in which motor and non-motor signs and symptoms were assessed as described later.

### Patients' Clinical and Functional Status Assessments

After consent to participate, patients were evaluated before and 1 year after DBS surgery with motor scales and a series of non-motor scales that include mood and pain evaluations. For the motor assessment, we registered the motor subscale (0–120) of the Burke–Fahn–Marsden Rating Scale (BFMRS). For mood and general non-motor symptoms, the Hospital Anxiety and Depression Scale (HADS) and the Non-Motor Symptoms Scale for Parkinson's Disease (NMSS-PD) were applied, respectively. The Parkinson's Disease Questionnaire-8 (PDQ8), derived from the Parkinson's Disease Questionnaire-39, was used to assess the quality of life (QoL). The questionnaires used for pain assessment included.

i) The Brief Pain Inventory (BPI) short form, which provides two principal scores: a pain severity score (mean of questions 3–6, items about pain intensity, each ranging between 0 and 10), and a pain interference score in daily activities (mean of questions 9A to 9G, each ranging from 0 to 10, and a sum pain interference score ranging from 0 to 70);

ii) Neuropathic Pain Symptom Inventory (NPSI), which also evaluates different clusters of descriptors of neuropathic and varies from 0 to 10 (total score is the sum of the 10 descriptors: 0–100) with two additional items related to the duration and frequency of paroxysmal pain;

iii) The short-form McGill Pain Questionnaire (MPQ) in which pain descriptors are categorized into three dimensions of pain: sensory (questions 1–8), affective (questions 9–13), and evaluative (questions 14–15). Also, there is an item for pain intensity by the visual analog scale (VAS, 0–100 mm, where 0 means no pain and 100 stands for maximal pain imaginable).

### Statistical Analysis

Data were expressed as mean ± SD (min–max). Non-normal data were evaluated using the Wilcoxon signed-rank test for dependent (i.e., paired) samples. The primary outcome was the change in BFMRS, HADS, and NMSS-PD scores and subscores 12 months after DBS surgery from the baseline condition. Secondary outcomes included the following: (1) these scores of patients with or without pain in the baseline condition (p_baseline_); (2) these scores of patients with or without pain after 12 months with DBS (p_1year_); (3) these scores of patients with pain in the baseline and after 12 months with DBS (p_withpain_); (4) these scores of patients without pain in the baseline and after 12 months with DBS (p_w/opain_); (5) the pain scales (e.g., BPI, NPSI, and MPQ) scores/subscores in the baseline condition and after 12 months with DBS. Spearman's rank correlation assessments were performed to verify whether changes (Δ = 12 months – baseline) in the different scales scores trended similarly or distinctively.

All statistical calculations were performed using the Statistical Package for the Social Sciences software (SPSS, version 27.0.0; SPSS Inc., Chicago, IL, USA), and statistical significance was set at *p* < 0.05. All datasets in this work are available from the corresponding author on reasonable request.

## Results

### Sample

**Figure 1:**
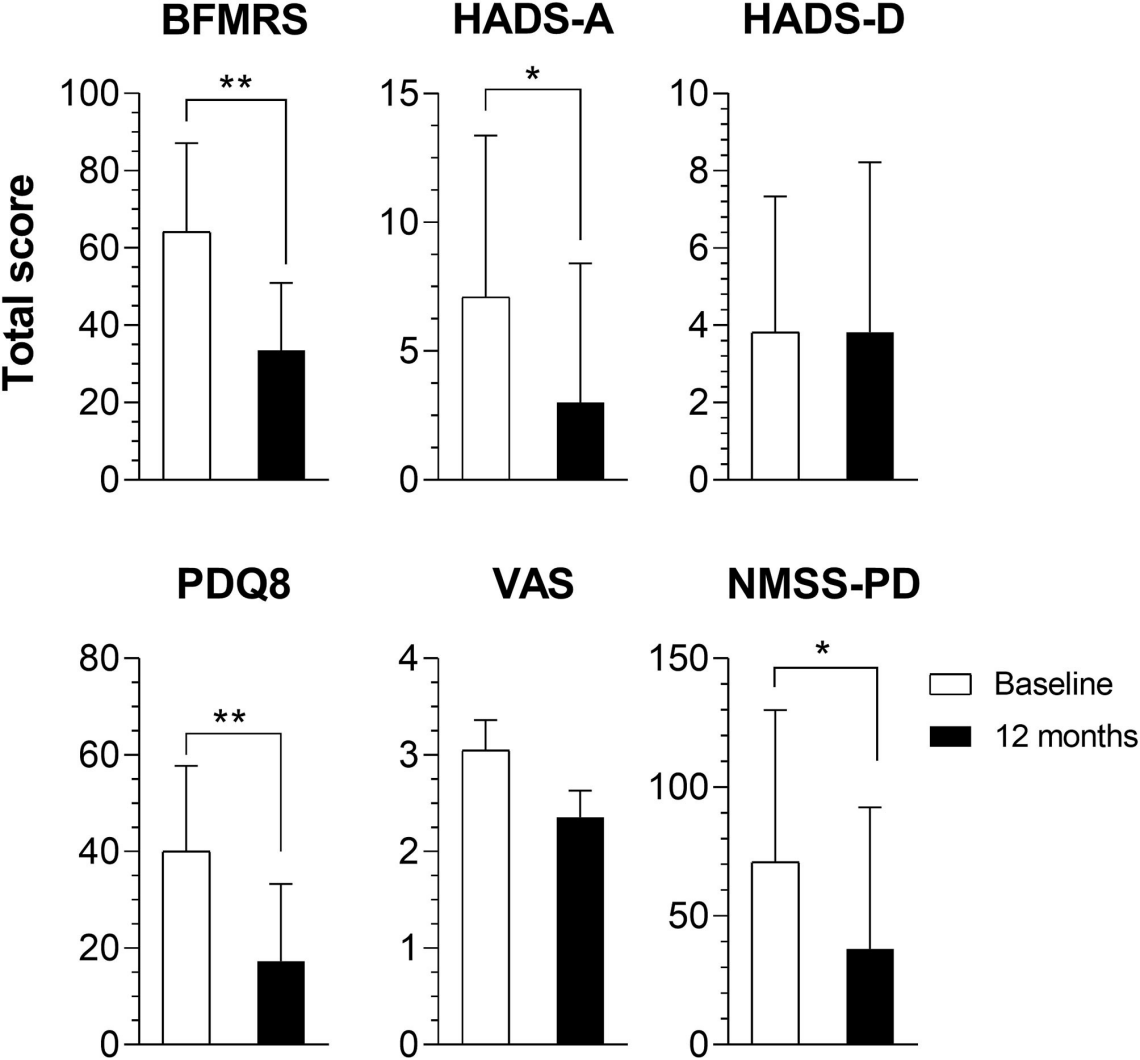
Comparison of baseline and 1-year total scores. Results of the Burke–Fahn–Marsden Rating Scale (BFMRS), Hospital Anxiety and Depression Scale (HADS) depression (HADS-D) and anxiety (HADS-A) subscores, Parkinson's Disease Questionnaire-8 (PDQ8), Non-Motor Symptoms Scale for Parkinson's Disease (NMSS-PD), and Visual Analog Scale (VAS) results of all patients. ^*^p < 0.05; ^**^p < 0.01.

All 11 unrelated patients underwent surgery. Age at surgery was 38.35 ± 11.30 (57.01–18.07) years (36.3% women). Four patients had DYT-*THAP1*, two DYT-*PRKRA*, and the others did not undergo genetic analysis (Supplementary Table 1). Of the entire cohort of patients, eight patients had GPi, one STN, and two STN-Substantia nigra (SN) as DBS targets. Motor BFMRS subscore was 64.36 ± 22.94 (31.00–102.00) at baseline and 33.55 ± 17.44 (9.00–58.50) 1 year after DBS surgery (*p* = 0.003, Table 1), which corresponds to a 47.9% improvement in motor symptoms related to dystonia (Figure 1).

**Table 1:** BFMRS, HADS, and NMS scale results.

**Scales**		**Baseline**	**1 year**	***p***
HADS	BFMRS (0–120)	64.36 ± 22.94 (31.00–102.00)	33.55 ± 17.44 (9.00–58.50)	0.003^**^
	Anxiety subscore (0–21)	7.09 ± 6.28 (0.00–20.00)	3.00 ± 5.40 (0.00–19.00)	0.028^*^
	Depression subscore (0–21)	3.82 ± 3.52 (0.00–12.00)	3.82 ± 4.40 (0.00–16.00)	0.856
	Total score (0–42)	10.91 ± 8.93 (0.00–32.00)	6.82 ± 9.66 (0.00–35.00)	0.102
	PDQ8 (0–100%)	(40.06 ± 17.72)% (9.38–62.50)%	(17.33 ± 15.96)% (0.00–43.75)%	0.005^**^
NMSS-PD	NMS Cardiovascular (0–24)	3.36 ± 4.06 (0.00–12.00)	3.82 ± 4.96 (0.00–14.00)	0.766
	NMS Sleep/Fatigue (0–48)	12.45 ± 10.13 (0.00–28.00)	9.27 ± 10.77 (0.00–32.00)	0.064
	NMS Mood/Cognitive (0–72)	12.27 ± 21.59 (0.00–72.00)	7.00 ± 14.87 (0.00–48.00)	0.161
	NMS Perceptual Problems/Hallucinations (0–36)	0.45 ± 1.04 (0.00–3.00)	0.00 ± 0.00 (0.00–0.00)	0.180
	NMS Attention/Memory (0–36)	9.45 ± 10.95 (0.00–28.00)	5.27 ± 6.94 (0.00–20.00)	0.075
	NMS Gastrointestinal (0–36)	14.27 ± 9.23 (0.00–28.00)	5.64 ± 8.98 (0.00–24.00)	0.041^*^
	NMS Urinary (0–36)	1.27 ± 3.61 (0.00–12.00)	0.73 ± 2.41 (0.00–8.00)	0.180
	NMS Sexual Function (0–24)	4.18 ± 7.72 (0.00–24.00)	2.18 ± 4.14 (0.00–12.00)	0.416
NMS Miscellaneous	Pain (0–12)	1.27 ± 3.61 (0.00–12.00)	1.09 ± 3.62 (0.00–12.00)	0.317
	Taste (0–12)	2.91 ± 5.09 (0.00–12.00)	0.00 ± 0.00 (0.00–0.00)	0.102
	Weight (0–12)	1.55 ± 3.56 (0.00–12.00)	0.00 ± 0.00 (0.00–0.00)	0.066
	Sweat (0–12)	7.45 ± 5.66 (0.00–12.00)	2.18 ± 4.85 (0.00–12.00)	0.024^*^
	Total (0–48)	13.18 ± 10.21 (0.00–32.00)	3.27 ± 7.76 (0.00–24.00)	0.012^*^
	NMS total score (0–360)	70.91 ± 59.07 (0.00–223.00)	37.18 ± 55.05 (0.00–176.00)	0.013^*^

*Data are presented as mean ± SD (min–max), in which sample size is n = 11*.

* *p < 0.05*;

** *p < 0.01 according to Wilcoxon non-parametric test. BFMRS, Burke–Fahn–Marsden rating dystonia scale; HADS, Hospital Anxiety and Depression Scale; NMS, non-motor symptoms; PDQ8, Parkinson's Disease Questionnaire 8; NMSS-PD, Non-Motor Symptoms Scale for Parkinson's Disease*.

### Mood and QoL

Total HADS scores did not change after DBS, ranging from 10.91 ± 8.93 (0.00–32.00) at baseline to 6.82 ± 9.66 (0.00–35.00) at 1 year with DBS (*p* = 0.102). However, the anxiety subscore had a significant decline, from 7.09 ± 6.28 (0.00–20.00) to 3.00 ± 5.40 (0.00–19.00); *p* = 0.028. QoL had a substantial improvement after DBS with PDQ8's baseline scores being 40.06 ± 17.72% (9.38–62.50%) and improving up to 17.33 ± 15.96% (0.00–43.75%) after 1 year (*p* = 0.005), an improvement of 56.7% (Table 1).

### Pain and Other NMS

Total NMSS-PD had a significant change 12 months after DBS, from 70.91 ± 59.07 (0.00–223.00) to 37.18 ± 55.05 (0.00–176.00), *p* = 0.013, a 47.5% improvement (Table 1). This was mainly driven by changes in the gastrointestinal (*p* = 0.041) and miscellaneous domains of the scale (*p* = 0.012).

Seven patients (63.6%) reported chronic pain (CP) before surgery (Supplementary Table 2), while only four patients reported it at 1 year postoperatively (i.e., pain improved in 42.3%). One year after DBS, BPI's severity and interference scores were 4.61 ± 2.84 (0.00–7.50) and 4.12 ± 2.67 (0.00–8.43), respectively, before surgery, and 2.79 ± 2.31 (0.00–6.25) and 1.12 ± 1.32 (0.00–3.00), respectively, after DBS. Both scores significantly improved in comparison to baseline (*p* = 0.043 and *p* = 0.028, respectively).

NPSI total score was 15.29 ± 13.94 (0.00–40.00) before DBS, while it was reduced to 2.29 ± 2.98 (0.00–7.00), *p* = 0.028 at 1 year. Likewise, MPQ total score was 9.00 ± 3.32 (3.00–12.00) before surgery, being reduced to 2.71 ± 2.93 (0.00–7.00), *p* = 0.028 after surgery. The comparisons of BFMRS, PDQ8, NMSS-PD, and HADS scores between both groups of patients at the baseline and 12 months after DBS did not significantly differ (Supplementary Table 3). QoL improved mostly in patients with pain after 1 year, 77.8% (*p* = 0.043). The BFMRS motor subscore also significantly improved, up to 48.7% (*p* = 0.043), in the subset of patients with pain.

The only differential scores that were significantly different from baseline and that had significant correlations were the PDQ8 and the NMSS-PD scales (ρ = 0.740, *p* = 0.009, Supplementary Figure 1). These results suggest that after 12 months, QoL and NMS scores rise significantly and correlate, suggesting that DBS's application improves NMS, which correlates with better QoL outcomes.

## Discussion

This is an original report on a comprehensive NMS assessment, including established pain scales, after DBS in refractory generalized inherited/idiopathic dystonia. Chronic pain was less reported 1 year after DBS, and all three pain scales improved after DBS. Our motor and QoL outcomes are in line with the literature (3). A systematic review found an improvement of pain in this same group of patients after GPi-DBS. Still, it emphasizes that no study has systematically looked into the correlation between motor and pain outcomes (8) or described the impact of DBS in the different dimensions of pain. Previous reports assessed pain using unidimensional tools such as the VAS, and it remains unknown the chronic pain state of patients at baseline. (8). In our sample, seven patients had chronic pain before DBS, which was reduced to four patients after 1 year of surgery. All pain scales we used detected statistically significant improvements after surgery.

Dystonia is a network disorder that involves both the basal ganglia-thalamo-cortical circuit and the cerebellum-thalamo-cortical circuit (11). Understanding its pathophysiology is still evolving, but it has been acknowledged that in dystonia there exists reduction of cortical inhibition, impaired synaptic plasticity, and sensory processing dysfunction (12). Also, sensory thresholds (13) and pain modulatory systems are altered in dystonia (14). The basal ganglia (BG) also plays an important function in pain processing (15) and other NMS processes such as mood and cognition (15). CP is characterized by multiple alterations in sensory, affective, and cognitive systems and impairment of modulatory systems that the BG is part of. Stimulation of both GPi (8) and STN has been reported to improve pain in many different conditions, including dystonia (8), probably due to different loop/pathway modulation (8), among other mechanisms.

Other non-motor symptoms have rarely been assessed. The NMSS-PD evaluates different NMS like cardiovascular/falls, sleep/fatigue, mood/cognition, hallucinations/perceptual problems, attention/memory/gastrointestinal tract (GIT), urinary, sexual function, and miscellaneous symptoms. NMS has significantly improved in our sample; interestingly, mainly driven by the GIT and miscellaneous subscores. Previous reports have shown an improvement in QoL; our cohort used PDQ8 with almost 78% improvements.

An important fact is that QoL improvement was mainly driven by NMS's improvement, while the improvement of the motor symptoms did not influence QoL in our correlation.

Despite their prevalence and impact on QoL, other NMS have rarely been assessed. The NMSS-PD evaluates different NMS and NMS have significantly improved in our sample. Previous reports have shown an improvement in QoL; our cohort used PDQ8 with almost 78% improvements.

Our study has some limitations, such as a small sample size with DBS being performed to different targets. However, generalized dystonia is a relatively rare disease, and most surgical studies reported on small sample sizes as well (3, 4). This is due to the rarity of the disease (only patients with idiopathic/inherited generalized dystonia included, and as expected with heterogeneous genetic subtypes). We have included two patients with DYT-*PRKRA*. There were two particular phenotypes in the original published DYT-*PRKRA* cases: (1) generalized isolated (pure) dystonia; (2) dystonia–parkinsonism dystonia that was non-responsive to levodopa (16). Therefore, as in other genetic diseases, one genotype can give rise to more than one phenotype. Many reports in the literature describe DYT-*PRKRA* patients as a form of isolated dystonia without parkinsonism (17). This example reported seven patients who did not have parkinsonism (four with generalized dystonia, one with focal dystonia, one with segmental dystonia, and one with multifocal dystonia). In the present study, the two DYT-*PRKRA* patients had isolated generalized dystonia phenotype, thus fulfilling our inclusion criteria (i.e., generalized inherited dystonia).

STN and GPi DBS may globally activate different cortical and subcortical networks in PD and dystonia. However, it may provide similar endpoints on motor symptoms of dystonic patients (6, 7). This similarity in motor outcomes may be due to

a) The activation of a group of common structures/hubs modulated by both approaches that provide a common and similar symptomatic relief by shared mechanisms;

b) Other variables. For instance, the percentage of motor improvement may be similarly obtained by both targets in different patients, which are not necessarily overlapping. In addition, analogous to two antidepressants that may provide a 60% response rate each in controlling depression, the patients responding to each of the two drugs may not be necessarily the same, given that these two drugs act by different mechanisms.

In support of point (a) above, an interesting study (18) evaluated BOLD functional MRI activation in motor and non-motor networks, comparing STN vs. EN/GPi [the entopeduncular nucleus (EN), the non-primate analog of the primate GPi] DBS in large animals. They found that both STN and EN DBS significantly increased BOLD activation in the ipsilateral premotor cortex, primary motor cortex, primary somatosensory cortex, dorsolateral prefrontal cortex, head of the caudate, anterior cingulate cortex, and insular cortex with both STN and EN/GPi stimulation. Combined with previous findings, their data support the idea that DBS has a neuromodulatory effect, facilitating the basal ganglia–thalamocortical loop complex in modulating global neural activity in motor and non-motor circuits. In addition, they found that STN stimulation induced greater activation in the caudate and putamen than EN/GPi.

Concerning the effects of both DBS target on NMS, it remains widely unknown the exact and detailed effects of DBS in general on NMS, as well as the effects of individual targets on these same symptoms. Such an approach would require a controlled and larger study, which cannot be provided by our current article. Here, based on the assumption that both targets have similar motor symptoms (again, with the mechanistic subtilities mentioned previously), we have explored the NMS of both approaches in a long-follow-up and prospective design, using a very detailed assessment of NMS, even recurring to still non-validated questionnaires when necessary. In addition, we found original data on NMS control brought about by surgery, which need further exploration in controlled settings.

Our findings highlighted the improvement in chronic pain and other NMS, along with the known improvements in motor symptoms and QoL (19). Also, this was not a controlled study, and one cannot rule out the effects of other factors on quality of life in the postoperative period such as dystonia medication decrease and concomitant rehabilitation treatment. At the time this study started, no tools specifically designed and validated to assess NMS related to dystonia existed; this is the reason we employed the NMS scale validated for Parkinson's disease. It is a 30-item rater-based scale to assess a wide range of NMS and measures the severity and frequency of NMS across nine dimensions. Despite not being validated to non-PD scenarios, we believe it is relatively easy to understand and to score and provides direct estimations of the frequency, impact, and intensity of several NMS that are too frequent in dystonia. As an exploratory study, we believe that the scale provides interesting information to the field. Also, we used PDQ8 as a QoL measurement; again, it is validated for another movement disorder, Parkinson's disease, and not to dystonia, and we acknowledge that this limits more elaborated analysis. However, its main domains—mobility, activities of daily living, emotional well-being, stigma, social support, cognition, communication, and bodily discomfort—are universal, and we believe that it could be applied to patients with other disorders for a general QoL overview, although keeping in mind the limitation described previously. Indeed, we have shown that QoL improvement was mainly driven by NMS's improvement, while improving the motor symptoms did not influence QoL in our correlation analyses. There currently exists a specific NMS scaled for dystonic patients (20), and future studies will be able to have a general view of NMS in dystonia based on it. Nevertheless, fine-grained information on specific symptoms will still demand the use of specific questionnaires and scales as we did here for chronic pain.

In conclusion, we found that DBS improves NMS in generalized inherited/idiopathic dystonia, including CP, and it correlated with improvements in QoL after surgery.

## Data Availability Statement

The raw data supporting the conclusions of this article will be made available by the authors, without undue reservation.

## Ethics Statement

The studies involving human participants were reviewed and approved by Comissão de Ética para Análise de Projetos de Pesquisa do HCFMUSP. The patients/participants provided their written informed consent to participate in this study.

## Author Contributions

CL: responsible for data curation, formal analysis, investigation, methodology, writing the first draft, and reviewing it. RC: responsible for conceptualization, project administration, and reviewing draft. SC: data curation and project managing. EL: formal analysis, investigation, and reviewing draft. DA: data curation. NS: data curation and project managing supporting. VD and RG: data curation and methodology. JM and JA: data curation. EB and MT: conceptualization and reviewing the draft. DD: conceptualization, reviewing the draft, and project managing. All authors contributed to the article and approved the submitted version.

## Conflict of Interest

The authors declare that the research was conducted in the absence of any commercial or financial relationships that could be construed as a potential conflict of interest.

## Publisher's Note

All claims expressed in this article are solely those of the authors and do not necessarily represent those of their affiliated organizations, or those of the publisher, the editors and the reviewers. Any product that may be evaluated in this article, or claim that may be made by its manufacturer, is not guaranteed or endorsed by the publisher.

## Supplementary Material

Supplementary Figure 1 NMSS-PD total scale correlation to the quality of life improvement. Spearman correlation analysis, the only variable that revealed significant correlation was the QoL's improvement to the NMSS-PD total scale.

Supplementary Table 1 Clinical information for patients with the study.

Supplementary Table 2 BPI, NPSI and MPQ scales scores in patients with or without pain. Data are presented as mean ± standard deviation (min–max), in which sample size is *n* = 4–7.

Supplementary Table 3 BFMRS, HADS and NMS scales scores in patients with or without pain. Data are presented as mean ± standard deviation (min–max), in which sample size is *n* = 4–7.
