# Supplementary figures and images for: Improvement of Non-motor Symptoms and Quality of Life After Deep Brain Stimulation for Refractory Dystonia: A 1-Year Follow-Up
Source: Front Neurol. 2021 Oct 4;12:717239. doi: 10.3389/fneur.2021.717239 (PMC8520898; doi:10.3389/fneur.2021.717239)

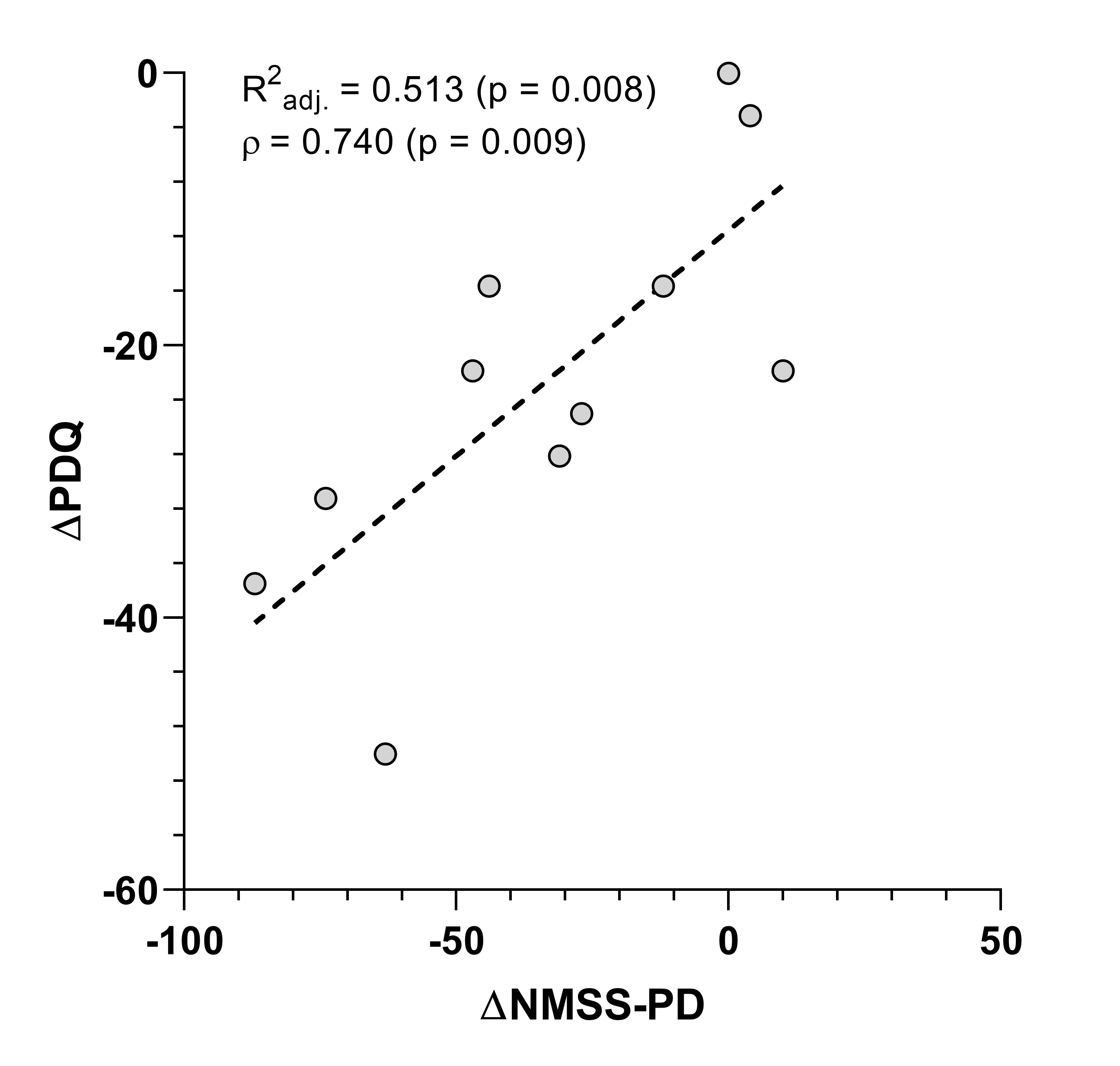

Supplement: Supplementary Figure 1 — NMSS-PD total scale correlation to the quality of life improvement. Spearman correlation analysis, the only variable that revealed significant correlation was the QoL's improvement to the NMSS-PD total scale. [file Image_1.TIF]
