# Supplementary material for: Improvement of Non-motor Symptoms and Quality of Life After Deep Brain Stimulation for Refractory Dystonia: A 1-Year Follow-Up
Source: Front Neurol. 2021 Oct 4;12:717239. doi: 10.3389/fneur.2021.717239 (PMC8520898; doi:10.3389/fneur.2021.717239)
Supplement: Supplementary Table 1 — Clinical information for patients with the study. [file Data_Sheet_1.PDF]

Supplementary File

Supplementary Table 1. Clinical information for patients with the study.

Number	Surgery Date	Sex	Age (yr)	DBS target	Duration of disease (yr)	Medication	Clinical aspects	Genetic analysis
1	feb/16/2016	F	40	GPi	10	6 mg/day clonazepam; 20 mg/day baclofen; ciclobenzaprine; venlafaxine	Generalized dystonia	N/A
2	may/30/2017	F	44	GPi	12	baclofen, carbamazepine, imipramine		N/A
3	jun/23/2017	M	41	GPi	37	30 mg/day diazepam; 2 mg/day biperiden; 30 mg/day baclofen	Generalized dystonia	N/A
4	jun/02/2017	M	27	GPi	5	baclofen, biperiden, clonazepam, ciclobenzaprine	Generalized dystonia	DYT-*THAP1*
5	jun/07/2017	M	32	GPi	27	60 mg/day baclofen; 2 mg/day clonazepam	Generalized dystonia	DYT-*THAP1*
6	oct/30/2016	M	25	GPi	8	30 mg/day baclofen	Generalized dystonia	DYT-*THAP1*
7	nov/07/2017	F	17	GPi	9	None	Generalized dystonia without parkinsonism	DYT-*PRKRA*
8	mar/23/2016	M	56	STN	46	ibuprofen, alprazolam	Generalized dystonia	N/A
9	jul/07/2016	M	37	STN	31	30 mg/day baclofen; 6 mg/day clonazepam	Generalized dystonia without parkinsonism	DYT-*PRKRA*
10	apr/06/2018	F	50	STN-SN	39	None	Generalized dystonia	N/A
11	feb/01/2018	M	43	STN-SN	34	None	Generalized dystonia	DYT-*THAP1*

M: Male; F: Female, GPi: Globus Pallidus internus; STN: Subthalamic nucleus; STN-Substantia nigra

Supplementary Table 2. BPI, NPSI and MPQ scales scores in patients with or without pain. Data are presented as mean ± standard deviation (min–max), in which sample size is n = 4–7.

Scales		***Baseline* (n = 7)**	**1 year (n = 4)**	**p**
BPI	Pain severity score (0–10)	4.61 ± 2.84 (0.00–7.50)	2.79 ± 2.31 (0.00–6.25)	0.043*
	Pain interference score (0–10)	4.12 ± 2.67 (0.00–8.43)	1.12 ± 1.32 (0.00–3.00)	0.028*
	NPSI (0–100)	15.29 ± 13.94 (0.00–40.00)	2.29 ± 2.98 (0.00–7.00)	0.028*
MPQ	Sensory (0–8)	3.36 ± 2.84 (0.00–8.00)	1.86 ± 1.35 (0.00–4.00)	0.026*
	Affective (0–5)	4.00 ± 0.00 (4.00–4.00)	2.00 ± 1.53 (0.00–4.00)	0.786
	Evaluative (0–2)	1.57 ± 0.54 (1.00–2.00)	0.57 ± 0.79 (0.00–2.00)	0.053
	Total score (0–15)	9.00 ± 3.32 (3.00–12.00)	2.71 ± 2.93 (0.00–7.00)	0.028*

*p < 0.0536 according to Wilcoxon non-parametric test. BPI: Brief pain inventory; NPSI: neuropathic pain symptom inventory;MPQ: Short-form McGill pain questionnaire.

Supplementary Table 3. BFMRS, HADS and NMS scales scores in patients with or without pain. Data are presented as mean ± standard deviation (min–max), in which sample size is n = 4–7.

Escalas			*Baseline*		$p_{baseline}$	1 year		$p_{1\ year}$	$P_{w/o\ pain}$	$P_{with\ pain}$
			**Pain (n = 7)**	**No pain (n = 4)**		**Pain (n = 5)**	**No pain (n = 6)**			
	BFMRS (0–120)		67.79 ± 23.60 (32.50–102.00)	58.40 ± 23.80 (31.00–88.00)	0.527	34.80 ± 22.40 (9.00–58.50)	33.25 ± 14.34 (13.00–54.00)	0.931	0.180	0.043*
HADS	Anxiety subscore (0–21)		8.86 ± 7.01 (2.00–20.00)	4.00 ± 3.65 (0.00–8.00)	0.230	1.40 ± 1.14 (0.00–3.00)	4.33 ± 7.26 (0.00–19.00)	0.662	0.655	0.068
	Depression subscore (0–21)		4.29 ± 3.68 (1.00–12.00)	3.00 ± 3.56 (0.00–8.00)	0.412	2.40 ± 1.82 (0.00–5.00)	5.00 ± 5.69 (1.00–16.00)	0.537	0.655	0.480
	Total score (0–42)		13.14 ± 10.22 (3.00–32.00)	7.00 ± 4.97 (0.00–11.00)	0.412	3.80 ± 2.86 (0.00–8.00)	9.33 ± 12.79 (1.00–35.00)	0.537	0.655	0.144
	PDQ8		45.09 ± 17.20 (21.88–62.50)	31.25 ± 17.11 (9.38–50.00)	0.315	10.00 ± 11.13 (0.00–28.10)	23.45 ± 17.65 (0.00–43.80)	0.329	0.180	0.043*
NMSS-PD	NMS Cardiovascular (0–24)		3.43 ± 3.21 (0.00–8.00)	3.25 ± 5.85 (0.00–12.00)	0.648	4.20 ± 5.67 (0.00–14.00)	3.50 ± 4.81 (0.00–12.00)	0.662	0.317	0.715
	NMS Sleep/Fatigue (0–48)		16.14 ± 10.17 (0.00–28.00)	6.00 ± 6.93 (0.00–16.00)	0.109	8.40 ± 8.62 (0.00–22.00)	10.00 ± 13.08 (0.00–32.00)	0.931	0.317	0.066
	NMS Mood/Cognitive (0–72)		18.00 ± 25.77 (0.00–72.00)	2.25 ± 3.86 (0.00–8.00)	0.230	1.80 ± 2.68 (0.00–6.00)	11.33 ± 19.66 (0.00–48.00)	0.931	0.317	0.144
	NMS Perceptual Problems/Hallucinations (0–36)		0.714 ± 1.254 (0.00–3.00)	0.00 ± 0.00 (0.00–0.00)	0.527	0.00 ± 0.00 (0.00–0.00)	0.00 ± 0.00 (0.00–0.00)	1.000	1.000	0.317
	NMS Attention/Memory (0–36)		13.29 ± 11.87 (0.00–28.00)	2.75 ± 4.86 (0.00–10.00)	0.315	5.20 ± 5.22 (0.00–12.00)	5.33 ± 8.64 (0.00–20.00)	0.792	1.000	0.180
	NMS Gastrointetinal (0–36)		16.71 ± 7.13 (8.00–28.00)	10.00 ± 12.00 (0.00–24.00)	0.412	6.00 ± 9.59 (0.00–22.00)	5.33 ± 9.35 (0.00–24.00)	0.931	0.317	0.078
	NMS Urinary (0–36)		2.00 ± 4.47 (0.00–12.00)	0.00 ± 0.00 (0.00–0.00)	0.527	0.00 ± 0.00 (0.00–0.00)	1.33 ± 3.27 (0.00–8.00)	0.662	1.000	0.317
	NMS Sexual Function (0–24)		5.43 ± 9.29 (0.00–24.00)	2.00 ± 4.00 (0.00–8.00)	0.648	3.20 ± 5.22 (0.00–12.00)	1.33 ± 3.27 (0.00–8.00)	0.537	1.000	0.785
	NMS Miscellaneous	Pain (0–12)	2.00 ± 4.47 (0.00–12.00)	0.00 ± 0.00 (0.00–0.00)	0.527	0.00 ± 0.00 (0.00–0.00)	2.00 ± 4.90 (0.00–12.00)	0.662	1.000	0.317
		Taste (0–12)	1.14 ± 3.02 (0.00–8.00)	6.00 ± 6.93 (0.00–12.00)	0.315	0.00 ± 0.00 (0.00–0.00)	0.00 ± 0.00 (0.00–0.00)	1.000	0.317	1.000
		Weight (0–12)	2.00 ± 4.47 (0.00–12.00)	0.75 ± 0.96 (0.00–2.00)	0.788	0.00 ± 0.00 (0.00–0.00)	0.00 ± 0.00 (0.00–0.00)	1.000	0.317	0.317
		Sweat (0–12)	8.29 ± 5.22	6.00 ± 6.93	0.648	2.40 ± 5.37	2.00 ± 4.90	0.931	1.000	0.109

	(0.00–12.00)	(0.00–12.00)		(0.00–12.00)	(0.00–12.00)			
Total (0–48)	13.43 ± 10.81<br>(0.00–32.00)	12.75 ± 10.63<br>(0.00–26.00)	0.788	2.40 ± 5.37<br>(0.00–12.00)	4.00 ± 9.80<br>(0.00–24.00)	1.000	0.317	0.109
NMS Total score (0–360)	89.10 ± 64.10<br>(26.00–223.00)	39.00 ± 35.90<br>(0.00–87.00)	0.164	31.20 ± 39.40<br>(0.00–100.00)	42.20 ± 68.90<br>(4.00–176.00)	0.792	0.655	0.080

*$p < 0.05$. $p_{baseline}$ and p_{1year} according to Mann-Whitney; and $p_{w/o\ pain}$ and $p_{with\ pain}$ according to the Wilcoxon non parametric test. BFMRS: Burke-Fahn-Marsden dystonia rating scale; HADS: Hospital anxiety and depression scale; NMS: non-motor symptoms; PDQ8: Parkinson’s Disease Questionnaire 8; NMSS-PD: Non-motor symptoms scale for Parkinson’s Disease.
